# Hedgehog Signaling in the Maintenance of Cancer Stem Cells

**DOI:** 10.3390/cancers7030851

**Published:** 2015-08-11

**Authors:** Catherine R. Cochrane, Anette Szczepny, D. Neil Watkins, Jason E. Cain

**Affiliations:** 1 Centre for Cancer Research, Hudson Institute of Medical Research, Clayton, Victoria 3168, Australia; E-Mails: catherine.cochrane@hudson.org.au (C.R.C.); anette.szczepny@hudson.org.au (A.S.); 2 Department of Molecular and Translational Science, Monash University, Clayton, Victoria 3168, Australia; 3 The Kinghorn Cancer Centre, Garvan Institute of Medical Research, Darlinghurst, New South Wales 2010, Australia; E-Mail: n.watkins@garvan.org.au; 4 UNSW Faculty of Medicine, Randwick, New South Wales 2031, Australia; 5 Department of Thoracic Medicine, St Vincent’s Hospital, Darlinghurst, New South Wales 2010, Australia

**Keywords:** hedgehog signaling, cancer stem cells, tumourigenesis

## Abstract

Cancer stem cells (CSCs) represent a rare population of cells with the capacity to self-renew and give rise to heterogeneous cell lineages within a tumour. Whilst the mechanisms underlying the regulation of CSCs are poorly defined, key developmental signaling pathways required for normal stem and progenitor functions have been strongly implicated. Hedgehog (Hh) signaling is an evolutionarily-conserved pathway essential for self-renewal and cell fate determination. Aberrant Hh signaling is associated with the development and progression of various types of cancer and is implicated in multiple aspects of tumourigenesis, including the maintenance of CSCs. Here, we discuss the mounting evidence suggestive of Hh-driven CSCs in the context of haematological malignancies and solid tumours and the novel strategies that hold the potential to block many aspects of the transformation attributed to the CSC phenotype, including chemotherapeutic resistance, relapse and metastasis.

## 1. Introduction

Cancer is a term encompassing a broad spectrum of disease uniformly defined by uncontrolled growth and underpinned by genomic instability, leading to further genetic diversity and intratumoural cellular and functional heterogeneity. As a result, two mutually non-exclusive models have been proposed to account for this tumour heterogeneity: the stochastic or clonal evolution model and the cancer stem cell model [1]. The conventional stochastic model postulates that all cancer cells within a tumour adapt and evolve to produce genetically- and phenotypically-distinct tumourigenic cells [2]. In contrast, the cancer stem cell model suggests the existence of a small population of primitive tumour cells that share many properties with somatic stem cells and the capacity to evolve into all cell types within a tumour, termed cancer stem cells (CSCs) [3,4]. Despite an accumulation of experimental evidence supporting this latter model, strong debate has ensued over the existence of CSCs. This is, in part, attributed to controversy surrounding the cell of origin, lack of a universal CSC marker and the limitations of the experimental techniques used to isolate and functionally-characterize CSCs.

CSCs represent a fractional cell population that exhibits unlimited self-renewal potential, has the ability to give rise to all cell types within a tumour and is resistant to many traditional cancer therapies that affect the more differentiated tumour bulk cells [5]. Although the existence of CSCs remains highly controversial, the CSC hypothesis is of considerable clinical importance, potentially explaining tumour insensitivity to chemotherapies, disease progression and relapse. To date, the cellular mechanisms that regulate CSC maintenance are poorly understood, although mounting evidence has implicated key developmental signaling pathways, including Hedgehog, Wnt and Notch, whose roles in regulating embryonic and adult stem and progenitor cells are better defined [6].

Although critical during embryonic organogenesis and adult homeostasis following repair and injury [7,8], aberrant activation of the Hedgehog (Hh) pathway also controls multiple aspects of tumourigenesis [9]. Together with a major role in maintaining the self-renewing capacity of adult somatic stem cells [10,11], it is not surprising that the Hh signaling has been widely implicated in CSC function and maintenance. If CSCs are the driving force behind tumour maintenance and growth, then understanding the role that Hh plays in regulating CSCs is of vital importance. This review focuses on the evidence that exists in favour of the CSC model, specifically the role of the Hedgehog pathway in CSCs in a variety of haematological malignancies and solid tumours, and highlights the strategies that hold the potential to block many aspects of transformation attributed to the CSC phenotype, through inhibition of the Hh signaling pathway.

## 2. The Hedgehog Signaling Network

Hedgehog signaling involves a wide variety of cellular and molecular mechanisms, such as protein trafficking, protein-protein interactions, positive and negative feedback loops and post-translational modifications, including phosphorylation, lipidation and proteolytic cleavage. This enables tight regulation of Hh signaling in a temporally- and spatially-specific manner, a key requirement for tissue patterning, cell fate determination and self-renewal.

### 2.1. Hedgehog Biogenesis and Secretion

**Figure 1:**
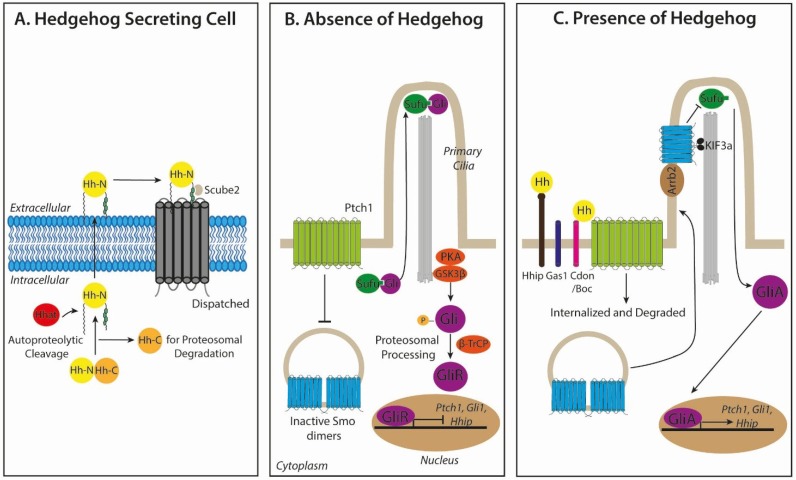
Mammalian Hedgehog (Hh) signaling. (**A**) Hh ligand precursor proteins are autoproteolytically cleaved to generate an N-terminal protein that undergoes dual lipid modification, consisting of an N-terminal palmitoyl group and a C-terminal cholesterol moiety, which promotes the binding of ligand to sterol-rich membrane microdomains to restrict mobility. The release of active Hh ligand is then mediated by Dispatched in synergy with Scube2. (**B**) In the absence of ligand at the responding cell, Patched 1 (Ptch1) constitutively inhibits Smoothened (Smo), preventing its ciliary localization. In this state, Gli proteins are retained in a complex with Suppressor of Fused (Sufu) at the ciliary tip. The recruitment of protein kinase A (PKA), glycogen synthase kinase 3β (GSK3β) and β-transducin repeat-containing protein (β-TrCP) to this complex results in partial proteasomal degradation to form Gli transcriptional repressors (GliR) that translocate to the nucleus and repress Hh target genes. (**C**) In the presence of the ligand, Hh binding to Ptch1 relieves repression of Smo, triggering its interaction with β-arrestin (Arrb2) and Kif3a and subsequent ciliary localization. This facilitates the release of Gli from Sufu, bypassing proteolytic cleavage into a repressor form, and full-length Gli activators (GliA) translocate to the nucleus to activate Hh target genes. High affinity Hh ligand-Ptch1 binding is facilitated by distinct multimolecular complexes with CAM-Related/Downregulated by Oncogenes (Cdon), Brother of Cdon (Boc) and Growth Arrest Specific 1 (Gas1). *Ptch1*, *Gli1* and *Hhip* are robust Hh target genes.

The three mammalian Hh ligands, Sonic Hedgehog (Shh), Indian Hedgehog (Ihh) and Desert Hedgehog (Dhh), are synthesized as precursor proteins that undergo autoproteolytic cleavage to produce an N-terminal signaling protein with dual lipid modifications [12,13] (Figure 1A). Cleavage of the carboxyl-terminal peptide and subsequent transfer of a cholesterol moiety on the resulting C-terminus leads to Hh ligand retention at the plasma membrane. Hedgehog acyltransferase (Hhat) catalyses the addition of a palmitoyl group on the N-terminus [14,15], promoting the association of the ligand to sterol-rich membrane microdomains to restrict ligand mobility [16,17]. Dispatched (Disp), a large multi-pass transmembrane protein, in synergy with Scube2, a secreted glycoprotein, bind to distinct components of the C-terminal cholesterol group to generate the release of Hh ligand from the plasma membrane and shelter lipidated Hh from the aqueous microenvironment [18,19]. Additionally, Hh contains the ability to form monomers and large multimers through their cholesterol linkages [20,21,22]. Diffusion of Hh ligand is negatively regulated by the membrane protein Hh-interacting protein 1 (Hhip1), which competes with the receptor Patched (see below) for ligand binding through association of the Zn^2+^ containing pseudo-active site in Hh ligands [23,24]. Similarly, the glycophosphatidylinositol (GPI)-linked heparan sulphate proteoglycan, Glypican-3, (Gpc3), is able to sequester Hh and prevent long-range ligand distribution [25,26,27].

### 2.2. Hedgehog Signal Transduction

Hh signaling is initiated by the binding of Hh ligand to its corresponding receptor, Patched (Ptch1, and to a lesser extent, Ptch2), a twelve-pass transmembrane protein located on Hh-responsive cells [28,29]. This process is also facilitated by co-receptors, CAM-Related/Downregulated by Oncogenes (Cdon), Brother of Cdon (Boc), and Growth Arrest Specific 1 (Gas1), which form distinct multimolecular complexes with Ptch1 to promote high-affinity Hh ligand binding [30,31]. In the absence of Hh ligand, Ptch1 constitutively represses Smoothened (Smo), a seven-transmembrane domain receptor of the G-protein-coupled receptor (GPCR) superfamily, preventing the translocation of Smo to primary cilia [32,33] (Figure 1B). Smo exists as inactive internalized dimers, where the cytoplasmic tails of each monomer are in a closed configuration maintained by electrostatic forces between arginine and asparagine clusters at the C-terminus [33,34,35]. In the absence of active Smo in the ciliary membrane, the Glioma-associated oncogene (Gli) family of latent zinc-finger transcriptional mediators, Gli1, Gli2 and Gli3, are retained in a complex with the negative regulator, Suppressor of fused (Sufu), at the ciliary tip [36,37]. In this state, Gli2 and Gli3 are phosphorylated by Protein kinase A (PKA) [38] and Glycogen synthase kinase 3β (GSK3β), creating a binding site for the adaptor protein β-transducin repeat containing protein (β-TrCP) [39]. The Gli/β-TrCP complex becomes subject to ubiquitination mediated by the Cul1-based E3 ligase, resulting in partial proteasomal degradation to form transcriptional repressors (Gli2R and Gli3R), which translocate to the nucleus and repress Hh target genes [40]. Gli1 is unable to be processed in this way and only occurs as a full-length transcriptional activator [41].

In the presence of Hh ligand, Ptch1 relieves the basal repression of inactive Smo by neutralizing the electrostatic interactions between Smo dimers through G-protein coupled receptor kinase-2 (Grk2)-mediated phosphorylation of the adjacent domain in the C-terminus, promoting an open conformation of active Smo [42,43] (Figure 1C). Simultaneously, Ptch1 becomes internalized and degraded by lysosomes. Smo associates with β-Arrestin (Arrb2) [35,44] and the intraflagellar microtubule motor protein Kif3a, within the ciliary membrane, facilitating the release of full-length transcriptionally-active Gli proteins (GliA) from Sufu, thereby bypassing proteasomal proteolytic cleavage and processing [35,45]. GliA proteins then translocate to the nucleus and transcriptionally activate Hh target genes. Direct targets for GliA are the Hh pathway genes, *Gli1*, *Ptch1* and *Hhip*, positive and negative regulators of Hh signaling, promoting feedback loops to enhance or reduce the Hh response [46]. Whilst canonical Hh signaling culminates in Gli-mediated transcription, there is growing evidence for “non-canonical” Hh signaling mechanisms. In this case, signaling may occur via Hh signaling components in alternative ways to the canonical paradigm [47,48,49,50,51,52,53,54,55,56,57,58,59,60,61,62]. Since a role for non-canonical Hh signaling in CSC maintenance is yet to be elucidated, this review will focus on the canonical pathway.

## 3. Roles for Hedgehog Signaling in Cancer

### 3.1. Modes of Signaling in Hh-Pathway-Dependent Cancers

The Hh pathway plays a crucial role during organogenesis in the developing embryo, by orchestrating reciprocal communicative events between different cells and tissues. The effect of Hh signaling varies according to the receiving cell type, by directing either cell proliferation, cell fate determination, epithelial-to-mesenchymal transitions and the rearrangement of cells by motility and adhesion changes [63]. Therefore, it is not surprising that inappropriate activation of Hh signaling in the adult can contribute to the initiation, growth and maintenance of cancer. Active Hh signaling can also induce treatment failure in cancer patients, by limiting chemotherapeutic responses or by actively inducing more aggressive and therapy-resistant tumours.

**Figure 2:**
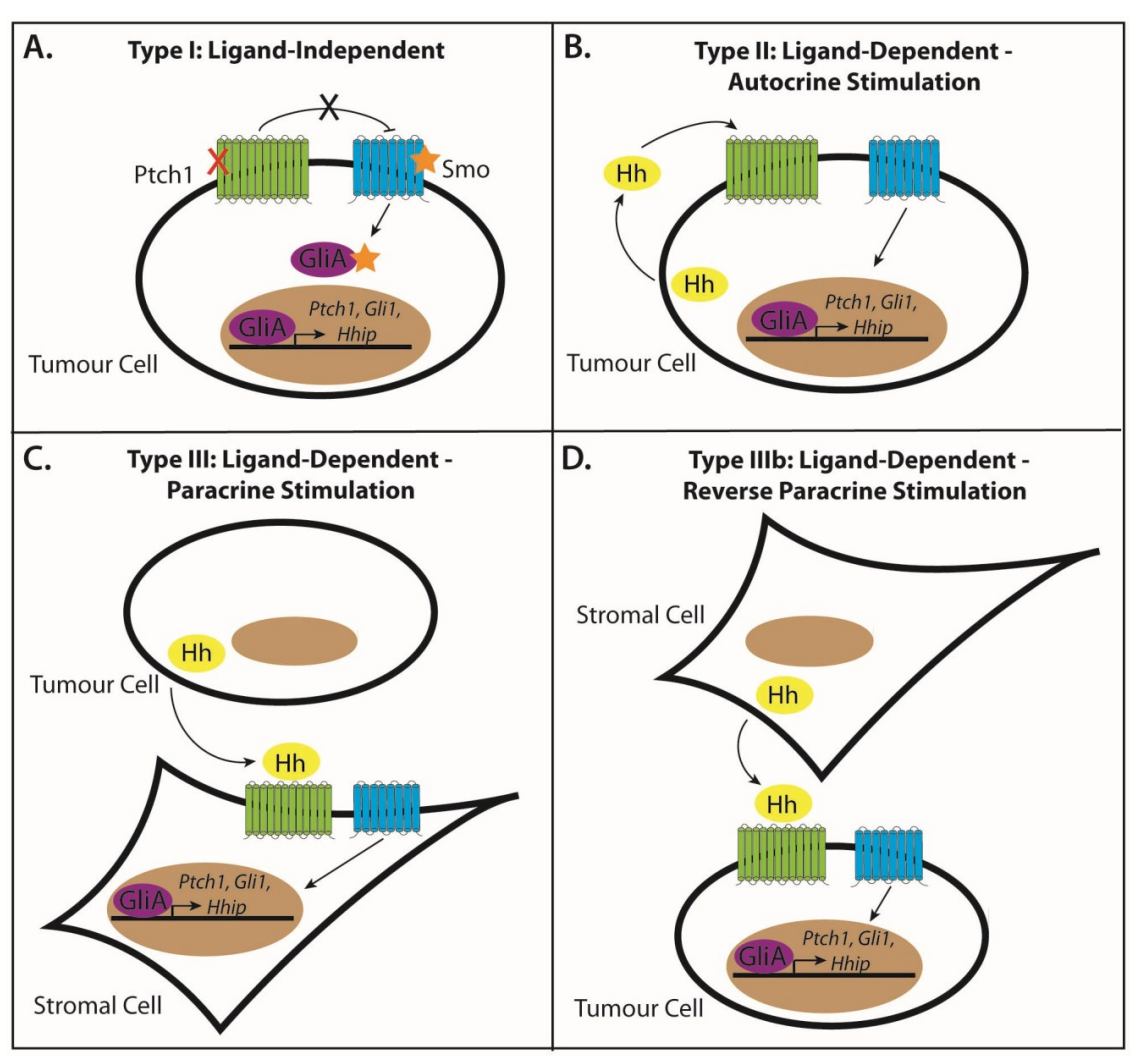
Modes of signaling in Hh pathway-dependent cancer. (**A**) Type I: ligand-independent, tumour cell-intrinsic signaling tumours exhibit mutations in the Hh pathway components that promote cell-intrinsic growth and survival. Loss of function mutations in Ptch1 (red cross), activating mutations of Smo and GliA amplifications (yellow stars), are common in these tumours. (**B**) Type II: ligand-dependent, autocrine stimulation is characterized by the response to the Hh ligand that is self-secreted. (**C**) Type III: ligand-dependent, paracrine signaling is defined by the secretion of the Hh ligand from the tumour cells that acts on adjacent stroma, in turn creating a favourable microenvironment for tumour growth. (**D**) In contrast, in Type IIIb ligand-dependent, reverse paracrine signaling, the Hh ligand is secreted by the adjacent stroma and acts on the tumour cells.

**Figure 3:**
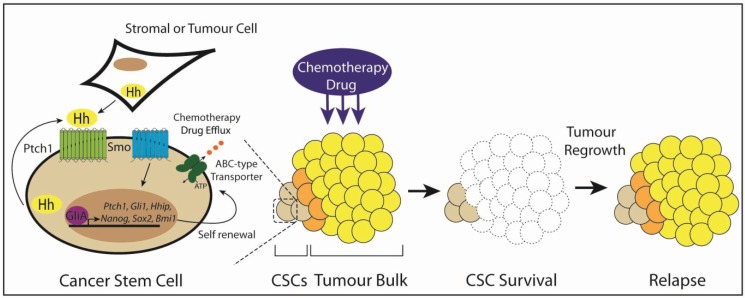
Hh signaling in cancer stem cells (CSCs). CSCs respond to the Hh ligand, secreted by adjacent stromal cells, tumour cells or the CSCs themselves, to maintain a stemness signature by the regulation of pluripotency genes, including *Nanog*, *Sox2* and *Bmi1*. CSCs are resistant to conventional chemotherapeutics, surviving treatment before expanding and deriving the heterogeneous tumour bulk population, resulting in disease relapse.

The major mechanisms by which the Hh pathway is aberrantly activated in cancer can be attributed to mutations of Hh pathway constituents (Type I: ligand-independent), excessive expression of Hh pathway ligands (Type II–IIIb: ligand-dependent) and the generation of a cancer stem cell (CSC) phenotype (Type IV) (Figure 2 and Figure 3). It is becoming increasingly apparent that it is essential to know which of these modes of signaling are in operation when evaluating experimental models of Hh-dependent cancer and also for considering the design of future tumour therapies involving Hh pathway inhibitors. Indeed, the use of the Hh pathway antagonists in clinical trials has shown promise in tumours driven by ligand-independent mechanisms, but so far has been underwhelming for those driven by ligand-dependent mechanisms. To further complicate matters, these signaling modes are not mutually exclusive and contain the ability to co-exist in parallel.

### 3.2. Type I: Ligand-Independent, Tumour Cell-Intrinsic Signaling

The association between Hh signaling and tumourigenesis was initially established in patients diagnosed with Gorlin syndrome, or nevoid basal cell carcinoma syndrome (NBCCS), where almost all cases are characterized by *PTCH1* loss of heterozygosity, leading to ligand-independent constitutive Hh pathway activation [64,65]. Tumours of type 1 origin have genetic aberrations in the Hh pathway components that promote cell-intrinsic growth and survival properties of the tumours (Figure 2A). Analysis of human cancer tissue and mouse models of Hh pathway activation have revealed that inactivating mutations, including deletions, mRNA splice-site and nonsense mutations in *PTCH1* [66], *SUFU* [67,68,69] or activating missense mutations in *SMO*, *SmoM2* (Trp535Leu) [70,71], or gene amplifications and translocations of *GLI1* or *GLI2* [72], usually in combination with the inactivation of additional tumour suppressor genes [73], are sufficient to form a variety of sporadic tumours [74]. This is especially the case for basal cell carcinomas (BCCs), a skin tumour of keratinocytes, medulloblastoma, a paediatric cancer of the cerebellum and rhabdomyosarcoma [75,76,77].

These findings implicate activating Hh pathway mutations as initiating events in tumourigenesis; therefore, Hh ligand independent tumours make excellent candidates for Hh pathway inhibitor therapy [78]. However, at what level of the signaling pathway a cancer cell has acquired such a pathway-activating genetic aberration will evidently determine whether, or not, a specific inhibitor is efficacious, as tumours with activating mutations downstream of SMO will be insensitive to the majority of Hh pathway inhibitors under development today.

### 3.3. Type II: Ligand-Dependent, Autocrine Signaling

The vast majority of tumours in which Hh signaling has been implicated lack mutations in the pathway and are dependent on upstream pathway activation driven by the Hh ligand. In this instance, tumour cells have been proposed to self-secrete Hh ligand in order to stimulate signaling, termed autocrine or juxtacrine Hh signaling [74,79] (Figure 2B). This was firstly based on tumour cells expressing both Hh ligand and downstream Hh signaling constituents, where growth was significantly inhibited by the naturally-occurring Smo antagonist, cyclopamine, in the absence of tumour stroma [80,81,82]. Whilst the non-specificity of cyclopamine initially cast some doubt over the interpretation of this finding [83], many more recent *in vitro* and *in vivo* studies using the Hh ligand-neutralizing antibody 5E1, RNAi-mediated knockdown of *SMO* or *GLI1*, GLI antagonists, such as GANT61, and treatment with various other specific small molecule SMO antagonists have demonstrated similar findings [84,85,86]. Therefore, tumours characterized by type II signaling are susceptible to Hh inhibition at either the level of Hh ligand, Smo or Gli.

### 3.4. Type III: Ligand-Dependent, Paracrine Signaling

During development, Hh signaling predominantly utilizes the paracrine mode of signaling whereby Hh ligands are produced and secreted by the epithelium to act on adjacent mesenchymal cells [87,88]. Therefore, it is conceivable to think that this mode of signaling would also be utilized to promote tumourigenesis. Indeed, emerging evidence suggests that several tumours, believed to utilize autocrine signaling, might instead, or in addition to this, function through paracrine effects on the adjacent stroma [89,90] (Figure 2C). The responding stroma, in turn, creates a favourable microenvironment that supports tumour growth, by supplying growth and survival factors in order to increase blood vasculature [91]. Human prostate, pancreatic, ovarian and colorectal cancers are thought to activate the Hh pathway via paracrine stimulation, and this response can be blocked by specific Hh inhibitors [92,93]. The precise paracrine feedback mechanisms thought to signal from the stroma to tumour cells remains to be elucidated. However, recent evidence suggests that the IGF and the Wnt signaling pathways are the likely candidates, as the insulin-like growth factor gene (*Igf1*), IGF pathway binding proteins and *Wnt* signaling molecules in the stroma were similarly modulated to *Gli1* and *Ptch1* in Hh inhibitor-treated tumour xenografts [90]. Therefore, it is probable that the stromal microenvironment responds to the Hh ligand secreted from tumour cells, to initiate the production and release of pro-angiogenic stromal feedback factors, promoting growth and survival signals back to the tumour [94,95].

### 3.5. Type IIIb: Ligand-Dependent, Reverse Paracrine Signaling

In this variant of paracrine signaling, stromal cells produce and secrete Hh ligands to influence tumour cells [89,96] (Figure 2D). Classified as type IIIb signaling, this mechanism of Hh signaling has been observed in haematological malignancies, including B-cell lymphomas, multiple myelomas and leukemias, in which Hh secreted from the bone marrow stroma is essential for the survival of cancerous B cells through the upregulation of the anti-apoptotic factor, Bcl2 [97,98]. Stromal Hh was also found in endothelial cells of high-grade, platelet-derived growth factor (PDGF)-induced gliomas [99]. Again, this tumour growth is impeded in response to the inhibition of the Hh pathway with either cyclopamine, 5E1 or the small molecule SMO antagonist, SANT-1 [97].

### 3.6. Type IV: Cancer Stem Cells

An alternative model to the Type I–III Hh pathway stimulation proposes that Hh signaling is important for the existence of a subpopulation of tumour cells that exhibit stem cell-like properties (Figure 3). This rare subset of tumour-initiating cells, termed cancer stem cells (CSCs), are proposed to maintain a self-renewing reservoir and differentiate into transient amplifying cells to produce a state of cellular heterogeneity within a tumour [4,5]. Hh signaling is believed to drive the CSC phenotype through the subverted regulation of stemness-determining genes. Indeed, Nanog, a transcription factor that acts as a master determinant of both embryonic stem cell self-renewal and the re-programming of differentiated somatic cells to pluripotency, is a direct transcriptional target of the Hh signaling pathway [100]. Furthermore, Hh signaling maintains a stemness signature in multiple cancers by driving the expression of stemness regulating genes, such as *Oct4*, *Sox2* and *Bmi1* [101,102,103]. While Hh-driven CSCs have been validated for numerous haematological malignancies, their existence in solid tumours remains more controversial. The emerging role for Hh signaling in the maintenance of both haematological malignancies and solid tumour CSCs is discussed in more detail below (also summarized in Table 1).

**Table 1:** Summary of evidence for the role of Hedgehog signaling in cancer stem cells.

Tumour Type	CSC Marker	Stemness Genes Expressed	Mode of Hedgehog Inhibition	Experimental Evidence	Combination Therapy	Refs.
Chronic Myeloid Leukaemia (CML)	CD34^+^, Lin^−^, Sca^+^, cKit^+^	-	Cyclopamine, Bcr-Abl infected *Smo*^−/−^ embryonic liver cells, Smo KO in CML mouse model, PF-04449913	14-fold reduction in CML LSCs, 60% of mice survived after 7 weeks	Cyclopamine and nilotinib, PF-04449913 and dasatinib	[104,105,106,107,108]
Acute Myeloid Leukaemia (AML)	-	-	IPI-926, PF-04449913, Cyclopamine, Endogenous Hhip, 5E1	Inhibits self-renewal and promotes myelomonocytic differentiation	Sorafenib and IPI-926, cyclopamine or Hhip or 5E1 and cytarabine	[109,110,111,112]
Acute Lymphoblastic Leukaemia(ALL)	-	*-*	Cyclopamine, IPI-926, KAAD-cyclopamine, SANT-1	Reduces long-term self-renewal in B-ALL, promotes apoptosis in T-ALL	-	[113,114]
Multiple Myeloma	CD138^neg^, CD19^+^	-	Cyclopamine, 5E1	Reduces CD138^neg^ self-renewal by inducing plasma cell differentiation	-	[98]
Glioma	CD133^+^, ALDH1^+^, ABCG2^+^	*NANOG, OCT4, SOX2*, *NESTIN*, *BMI1*	Cyclopamine	Abolishes tumour engraftment	Cyclopamine, temozolomide and/or 10 Gys of radiation	[101,115,116,117]
Breast Cancer	CD44^+^, CD24^−/low^, Lin^−^, ALDH1^+^	*p63*, *OCT4*, *NESTIN*, *NANOG*, *BMI1*	Cyclopamine	Reduces mammosphere self-renewal and secondary formation	-	[118,119,120]
Small Cell Lung Cancer	-	*BMP4*, *NESTIN*, *ASH-1*	Cyclopamine, LDE-225, *shSMO*, 5E1	Prevents tumour relapse in LX22 xenografts	LDE-225, carboplatin and etoposide or, GDC-0449 and cisplatin	[82,121,122]
Non-Small Cell Lung Cancer	-	*SOX2*, *OCT4, NANOG*, *ALDHA1*	*siSHH* GDC-0449	Decreases colony formation and growth in soft agar	GDC-0440, erlotinib and cisplatin	[103,122,123]
Gastric Cancer	CD44^+^, CD24^+^	*SOX2*, *NANOG*	Cyclopamine, Vismodegib, 5E1, *shSMO*	Reduces CD44^+^ tumourspheres and number and diameter of colonies	Vismodegib, 5-flurouracil and/or cisplatin or cyclopamine, oxaliplatin and mitomycin	[124,125]
Colon Cancer	CD133^+^	*NANOG*, *OCT4*	*shSMO* Cyclopamine	Reduction of the CD133^+^ CSC population	-	[83,102]
Pancreatic Cancer	CD44^+^, CD24^+^, ESA^+^	*NANOG*, *OCT4*	GDC-0449, Cyclopamine derivative - CyT	Reduces tumoursphere viability and chemoresistance	CyT and 2 Gys of radiation	[86,126,127,128,129,130,131,132]
Prostate Cancer	-	*NANOG*, *OCT4*	Cyclopamine, *shGLI1,2*, GANT61	Suppresses tumoursphere and colony formation	Cyclopamine and paclitaxel	[133,134,135,136]
Metastatic Melanoma	ALDH^+^	*SOX2*, *NANOG*, *OCT4*, *KLF4*	*shSMO, shGLI1*	Reduces ALDH^+^ melanospheres fraction, clonogenicity and xenograft growth	-	[137,138,139]

## 4. Evidence for the Role of Hedgehog Signaling in Cancer Stem Cell Maintenance

### 4.1. Leukemic Stem Cells

Much of our knowledge of CSC biology is derived from studies on normal and malignant haematopoiesis, which has led to the identification of hematopoietic stem cells (HSCs) and its malignant counterpart, the leukemic stem cell (LSC) [140,141]. The stem cell theory of cancer, which postulates that malignancy arises from the transformation of adult somatic stem cells, is an attractive hypothesis within the hematopoietic system, as evidence indicates that the cell surface phenotype, CD34^+^ CD38^−^, is shared between LSCs and HSCs [141,142,143]. In addition, cytogenetic abnormalities that are consistently associated with certain leukemias have been detected in HSC compartments in patients with acute myeloid leukaemia (AML), chronic myeloid leukaemia (CML) and acute lymphoblastic leukaemia (ALL) (see below) [144,145,146,147]. For instance, the *BCR-ABL* gene rearrangement in CML has also been detected in cells of myeloid, erythroid megakaryocytic and B-lymphoid lineages, indicating that initial transformation occurs within a cell that is capable of multi-lineage differentiation [142,144,148]. Moreover, a genetic mouse model displaying conditional gene inactivation of Jun-B in the HSC compartment symptomatically produces myeloproliferative CML-like disease [149]. These results support the notion of a normal HSC hierarchy in the LSC compartment and implicate the HSC as the candidate cell for transformation by leukaemia-inducing oncogenes. In contrast, the ability of both *MLL-ENL*, a t(11;19) translocation in infant acute leukemias, and *MOZ-TIF2*, an AML inversion of (8)(p11q13), fusion oncogenes to restore self-renewal ability to previously-committed progenitors that normally lack the capacity to self-renew, and evidence of AML LSCs derived from the CD34^−^ fraction [150] provide an alternative origin of the LSC [151,152].

### 4.2. Chronic Myeloid Leukaemia

Chronic myeloid leukaemia (CML) is the best understood hematologic and stem-cell driven malignancy. Characterized as a clonal myeloproliferative disease, CML is caused by a chromosomal translocation forming the Philadelphia chromosome, which encodes the constitutively-active oncogenic tyrosine kinase, BCR-ABL. The tyrosine kinase inhibitor (TKI) Imatinib, which pharmacologically blocks BCR-ABL kinase activity, has radically revolutionized the management of chronic-phase CML, inducing unprecedented cytogenetic and molecular responses in patients [153].

Despite this success, TKI resistance is still an ongoing problem, and discontinuation of Imatinib can promote relapse of the disease [153]. Hh signaling is intimately involved in the persistence and self-renewing ability of BCR-ABL-driven Lin^−/^Sca^+^/cKit^+^ LSCs. Genetic inactivation of *Smo* in a mouse model of CML decreases the number of CML LSCs, whereas constitutively-active Smo results in a four-fold increase in CML LSCs, leading to accelerated CML tumourigenesis [104]. Similar results have been described by the overexpression of Bcr-Abl in both *Smo*^−/−^ and *Ptch1*^−/*+*^ embryonic liver cells transplanted into lethally-irradiated C57BL/6 mice, leading to failed expansion and reduced CML incidence or increased cell expansion, respectively. These studies strongly suggest that Hh pathway activity controls the frequency and maintenance of CML leukemic stem cells (LSCs) and, consequently, the incidence and latency of CML development [105,154]. Indeed, the delivery of cyclopamine to mice transplanted with Bcr-Abl-infected HSCs produced a 14-fold reduction in the CML stem cell population, where 60% of the mice survived after seven weeks [105]. It has also been shown that CML LSCs are dependent on low levels of the cell fate determinant, Numb. Numb, which plays a role in the regulation of Gli1 via Itch-dependent ubiquitination, was found to be highly expressed in *Smo*^−/−^ CML LSCs. In addition, ectopically-expressed Numb inhibited the *in vitro* expansion of Bcr-Abl-infected hematopoietic cells and CML LSCs derived from leukaemia patients [104].

Smo overexpression in human CML cell lines is also associated with reduced expression of miR-326 in CD34^+^ CML LSCs, and overexpression of miR-326 leads to Smo downregulation and, consequently, decreased cell viability [106]. This suggests that inhibition of Smo might result in the restoration of Numb and miR-326 expression, could possibly eradicate the number of CD34^+^ LSCs and, thus, CML pathogenesis. Additionally, Hh signaling has also been identified as an essential component of multidrug resistance (MDR) in myeloid leukaemia. Cyclopamine treatment of chemo-resistant Lucena-1 cells, derived from the CML chemotherapy-sensitive cell line K562, leads to the downregulation of P-glycoprotein, a notoriously-known ATP-dependent efflux pump that removes cytotoxic drugs from cancer cells, resulting in resensitization to chemotherapy [107]. Interestingly, treatment of CML patient-derived bone marrow cells with a combination of cyclopamine and the Abl inhibitor, Nilotinib, *in vitro*, reduced the number of colony-forming units by more than 80%. Similar results were also observed using PF-04449913, an orally bioavailable small molecule Smo antagonist, and the TKI Dasatinib, which reduced CML LSC burden by Gli2 inhibition [108], suggesting that targeting both tyrosine kinase and Hh activity might be an effective combination therapy in CML patients [105].

### 4.3. Acute Myeloid Leukaemia

AML, characterized as a malignancy of the myeloid line of blood cells, is an extremely heterogeneous clonal disorder with a phenotypically-variable LSC population that is likely not confined to a single clonal subpopulation [155]. Primary AML cell lines express *SHH* and *GLI1*, the latter of which correlates with cytogenetic risk and overall reduced survival [156]. Constitutive tyrosine kinase activity involving internal tandem duplications (ITD) of the FMS-like tyrosine kinase 3 (FLT3) juxtamembrane domain are typically found in AML patients. Consequently, *GLI2* expression in *FLT3-*ITD is higher than in wild-type *FLT3* AML patients, which correlates with reduced survival [109].

Mice expressing *FLT3*-ITD and SmoM2 in the hematopoietic system driven by *Mx1-Cre* and poly(I:C) treatment produce accumulated populations of c-Kit^+^Gr-1^int^ and Mac1^+^Gr1^int^ blasts of the myeloid lineage, leading to myeloproliferative neoplasia (MPN)-AML [109]. Constitutive Hh activation leads to downstream STAT5 signaling, and combined treatment with the tyrosine kinase inhibitor, sorafenib and SMO antagonist IPI-926 inhibited clonogenic AML growth and proliferation in *FLT3*-ITD^+^ AML cell lines *in vitro* and disease progression *in vivo* [109]. Together, these studies suggest that active Hh signaling in the granulocyte/monocyte progenitor compartment, in combination with the *FLT3-*ITD mutation, is capable of initiating the development of AML. Furthermore, Hh pathway inhibition with PF-04449913 sensitizes AML chemoresistant cell lines and primary cells to standard chemotherapy drugs, inhibits Smo-mediated self-renewal [110] and promotes myelomonocytic differentiation in the AML cell line, HL-60 [111]. Similarly, the chemotherapy-resistant CD34^+^ AML cell lines, Kasumi-1, Kasumi-3 and TF-1, express *IHH*, *GLI1* and *GLI2* and respond to cyclopamine, endogenous HHIP and 5E1 treatment, which in combination with cytarabine (Ara-C), dramatically reduces cell survival [112]. Conversely, in an alternative AML mouse model driven by *MLL-AF9*, the most frequent rearrangement in childhood AML, Hh pathway blockade was ineffective, signifying that Hh signaling is dispensable in this particular molecular subtype [157,158]. Given these conflicting results, the complexity between the intrinsic and extrinsic signals that govern LSC behaviour, and the high phenotypic variability in AML, it is probable that the underlying genetic and molecular mechanisms likely determine the suitability of targeting the Hh pathway in AML LSCs [157,159].

### 4.4. Acute Lymphoblastic Leukaemia

Characterized by the accumulation of malignant white blood cells, or lymphoblasts, acute lymphoblastic leukaemia (ALL), is the most common form of cancer in children. Hh signaling plays a key role in regulating self-renewal of ALL tumour cells of both B- and T-cell origin. In a panel of primary B-ALL cell lines, 95% expressed *PTCH1*, *GLI1* and *SMO* [113]. Moreover, treatment with the Smo inhibitors, cyclopamine or IPI-926, significantly reduces long-term self-renewal potential in B-ALL LSCs [113,114]. In the human-derived T-ALL cell line, CEM, inhibition of Hh activity with KAAD-cyclopamine or SANT-1 treatment induced cellular apoptosis in both CEM-derived glucocorticoid (GC)-sensitive and resistant T-ALL clones, highlighting a critical role for the Hh pathway in cell growth and survival [160].

### 4.5. Multiple Myeloma 

Multiple myeloma (MM), a malignancy consisting of a well-defined stem cell compartment, is a plasma cell malignancy of the bone marrow characterized by abnormal proliferation of plasma cells. MM consists of two distinct populations, CD138^neg^ CD19^+^ stem cells, and malignant CD138^+^ CD19^neg^ terminally-differentiated plasma cells [161]. Preferential expression of *SMO* and a GLI-responsive YFP reporter in the CD138^neg^ fraction of the MM cell line, NCI-H929, suggests that Hh signaling plays an important role in the CD138^neg^ LSC population. Indeed, inhibition of Hh signaling by cyclopamine or 5E1 treatment inhibited the clonal capacity of MM cell lines, NCI-H929 and KMS12, and decreased the CD138^neg^ population through the induction of plasma cell differentiation, highlighting a crucial role in the maintenance of self-renewal [98].

Intriguingly, experimental data suggests multiple modes of Hh signaling seem to activate MM CD138^neg^ LSCs. Bone marrow biopsy samples from MM patients demonstrate that SHH is mainly secreted by malignant CD138^+^ terminally-differentiated plasma cells, utilizing type III paracrine Hh signaling, to promote proliferation and the inhibition of chemotherapy-induced apoptosis [162]. In addition, stromally-induced type IIIb Hh signaling can also activate Hh signaling in MM LSCs [97]. Taken together, MM demonstrates that Hh signaling can act through multiple signaling modes within the same cancer and can mediate interactions between CSCs, differentiated tumour cells and the microenvironment [163].

### 4.6. Glioma

Glioma, one of the most common and lethal primary brain tumours, contain cells with shared similarities to normal neural stem cells, capable of clonogenic growth *in vitro* and tumour formation *in vivo* [164]. Hh signaling appears to be active in glioblastoma multiforme (GBM)-derived neurospheres and glioma stem cell cultures (gliomaspheres), as they express *GLI1*, *PTCH1*, *SMO* and *SHH* [115,116]. Indeed, conditioned media from GBM neurospheres induces a 10-fold increase of Gli1-luciferase in the NIH 3T3-Light2 reporter cell line [115]. Conversely, GBM neurospheres upregulate *GLI1* mRNA in response to conditioned medium containing exogenous SHH-N, indicating that GBM neurospheres are capable of secreting and responding to biologically-active SHH ligand [115].

Hh pathway blockade in GBM neurospheres by cyclopamine reduces *GLI1* mRNA expression, inhibits cell growth and promotes the formation of well-differentiated GFAP-positive neurospheres, indicative of mature glial cells [115], and downregulated the expression of stemness genes *NANOG*, *OCT4*, *SOX2*, *NESTIN* and *BMI1* [101]. Remarkably, intracerebral implantation of viable cyclopamine-treated neurospheres into NOD/SCID mice completely abolishes tumour engraftment and growth, indicating that inhibition of Hh signaling impedes clonogenic growth and self-renewal of GBM stem cells (GSCs). Combination treatment of cyclopamine and 10 Gys of radiation [115] or temozolomide [101] revealed a synergistic effect to reduce neurosphere growth, implying that Hh blockade targets GSCs that are not normally affected by radiation and chemotherapy. Indeed, *SMO*, *GLI1*, *PTCH1*, CD133 and aldehyde dehydrogenase (ALDH)-expressing GSCs are upregulated post-radiation in GBM neurospheres, which further supports the concept that standard therapies are not able to abolish GSCs [115]. Additionally, it has been shown that active Hh signaling can sensitize GSCs to endogenous nano-irradiation, through the inhibition of thymidine synthesis [117].

### 4.7. Breast Cancer

After the initial demonstration of CSCs in hematopoietic malignancies, breast cancer was the first solid malignancy in which CSCs were identified and isolated [2,118]. The CSC population, characterized with the cellular identity CD44^+^/CD24^−/low^Lin^−^ALDH-1^+^ and the capacity to recapitulate the phenotypic heterogeneity of the primary tumour when injected into secondary NOD/SCID mice [6,165], has been demonstrated to display active Hh signaling to maintain stemness potential. Since Hh signaling plays a critical role in mammary stem cell maintenance within the mammary epithelium [166,167], it is not surprising that mammary CD44^+^/CD24^−/low^Lin^−^ CSCs express increased mRNA transcript levels of *PTCH1*, *GLI1* and *GLI2* compared to bulk tumour cells [168]. This supports the stem cell theory of cancer, where malignancy arises from the transformation of adult somatic stem cells to utilize existing stem-cell regulatory pathways to promote self-renewal.

The mechanism of Hh-mediated self-renewal in mammospheres has been shown via Shh-mediated upregulation of the polycomb gene *BMI1*, an effect that was blocked by treatment of cyclopamine [118]. Indeed, isolated CD44^+^/CD24^−/low^Lin^−^ CSCs express a five-fold increase in *BMI1* compared to tumour cells also derived from the same human breast carcinoma-derived xenograft tumour, but lacking CSC marker expression [118]. Furthermore, p63, the sister homolog of p53, characterized as a master regulator of normal epithelial stem cell maintenance, drives Hh signaling in mammary CSCs [119]. Knock down of p63 in mammospheres derived from breast tumours of transgenic mice with conditional overexpression of the *ErbB2* oncogene in mammary glands results in a decrease of *Shh*, *Ptch1*, *Gli2* and *Bmi1* transcript levels, leading to a reduction in secondary mammosphere formation. ChIP-sequence analysis in p63 overexpressing MCF-7 cells has demonstrated that *Shh*, *Gli2* and *Ptch1* are direct transcriptional target genes of p63 [119]. Additionally, Hh inactivation in MCF-7-derived CD44^+^/CD24^−^ CSCs induced a reduction in cell number through downregulation of *OCT4*, *NESTIN* and *NANOG*, indicating that Hh signaling in breast CSCs upregulates stem cell markers to maintain a self-renewing signature [120].

### 4.8. Gastrointestinal Cancers

Studies have demonstrated the presence of gastric CSCs in several gastric cancer cell lines. Identified by the cell surface marker CD44^+^ and the ability to form non-adherent spherical colonies in serum-free media and tumours when implanted into immunocompromised mice, CD44^+^ gastric CSCs represent ~0.6%–2.2% of the tumour cell population [169]. MGC-803, HGC-27 and MKN-45 tumourspheres display increased mRNA expression of Hh components *Shh*, *Ptch1* and *Gli1* and stemness markers *Sox2* and *Nanog* [124] compared to adherent cultures.

Inhibition of Hh signaling in tumourspheres with cyclopamine, 5E1, vismodegib or *shSMO* reduces the capacity for the formation of CD44^+^ sub-tumourspheres in culture, as well as the number and diameter of colonies derived from single cells on soft agar, but had no effect on adherent cells [125]. Furthermore, vismodegib treatment dramatically increases CD44^+^ tumoursphere sensitivity to 5-flurouracil or cisplatin with vismodegib, reducing cell viability by ~87%, compared to only 13%–20% and 11%–22% with 5-fluorouracil and cisplatin treatment alone, respectively [124]. Additionally, dissociated HGC-27 tumourspheres treated with cyclopamine, followed by oxaliplatin and mitomycin, significantly enhanced the overall rate of apoptosis compared to cyclopamine and drug treatment alone [125]. Similar effects have been observed *in vivo*, with *shSMO*-transduced MKN-45 tumoursphere-derived xenografts demonstrating reduced growth potential following cisplatin treatment, associated with a decrease in CD44 expression [124].

Colon CSCs are thought to originate from the few stem cells situated at the base of colonic crypts [83,170]. Recurrence and metastatic spread of colon carcinomas have been proposed to depend on CD133^+^ CSCs, which induce tumours when implanted into nude mice. Although initiated from constitutive activation of Wnt signaling, Hh signaling plays an important role in the maintenance of colon CD133^+^ CSCs, which display the highest gene expression levels of *GLI1*, *PTCH1*, *GLI2*, *SHH* and *HHIP* compared to all CD133^−^ cells in human colon carcinoma samples [83]. Serial *in vivo* passaging of purified CD133^+^ CSCs stably expressing *shSMOH* or *shPTCH1* demonstrated a complete abolishment or increase the CD133^+^ CSC population, respectively, highlighting a critical role for Hh signaling in the self-renewal of these cells [83]. Furthermore, increased expression of *GLI1* and *SMO*, as well as stemness markers *NANOG* and *OCT4* in the HCT-116 non-adherent spheres compared to adherent cultures are significantly reduced following cyclopamine administration [102].

### 4.9. Pancreatic Cancer

In pancreatic ductal adenocarcinoma, tumour cells with the CD44^+^CD24^+^ESA^+^ immunophenotype convey the properties of self-renewal and multilineage differentiation and are thus considered the pancreatic CSC population [171,172,173]. Pancreatic CSCs-tumourspheres derived from cell lines AsPC-1, PANC-1 and MIA-PaCa-2 and pancreatic-derived metastases from an orthotopic mouse model of pancreatic cancer displayed increased *mRNA* and protein expression of Smo, Gli1 and Gli2 [126,127,128]. Moreover, Hh pathway blockade by GDC-0449 [129], a small molecule Smo antagonist, and GANT61 [86], a Gli inhibitor, reduced cell viability and induced apoptosis via Fas, DR4 and DR5 expression in all pancreatic tumoursphere cultures.

Similar results were observed following cyclopamine treatment, as well as a reduction in the expression of Bmi1 and the ATP-binding drug transporter ABCG2, suggesting that Bmi1 may function as a downstream Hh target in pancreatic cancer, as in breast cancer, and Hh blockade can reverse chemoresistance via ABCG2 downregulation in pancreatic CSCs [127,130]. Additionally, 2 Gys of radiation in the presence of CyT, a cyclopamine derivative, completely eliminated the pancreatic tumoursphere population, compared to radiotherapy and CyT treatment alone [128]. Hh pathway blockade in pancreatic CSCs can also be induced by sulforaphane (SFN), a compound derived from cruciferous vegetables, and epigallocatehin-3-gallate (EGCG), an active compound in green tea, which downregulated mRNA expression of Hh pathway components *Smo*, *Gli1* and *Gli2*, and pluripotency transcription factors, *Nanog* and *Oct4*, inhibited Gli-luciferase reporter activity and reduced the expression of Snail, Slug and ZEB, factors involved in invasion and migration, which produced an overall anti-proliferative and increased apoptotic effect in pancreatic CSCs [126,131,132]. Thus, Shh-Gli signaling plays an essential role in controlling stemness and chemotherapeutic resistance in pancreatic CSCs.

### 4.10. Prostate Cancer

Specifically an androgen-dependent disease, prostate cancer (PaC) often responds to androgen deprivation therapy (ADT) [174]. In the event of PaC relapse and metastasis, first-line chemotherapy drugs, such as paclitaxel and docetaxel, microtubule stabilizers, prove to be effective until the occurrence of relapse and disease progression from highly-chemoresistant prostate CSCs (PCSCs) [133,175]. CSC-containing side populations exhibit a higher expression of pluripotency markers *OCT4*, *NANOG* and ABCG2 compared to the non-side population fraction [134]. Remarkably, inhibition of the Hh signaling pathway in chemoresistant PCSCs, by cyclopamine, in combination with paclitaxel has been shown to significantly reduce cell viability and enhance apoptosis, when compared to paclitaxel and cyclopamine treatment alone [134]. Similarly, inhibition of *GLI1* and *GLI2* in docetaxel-resistant prostate cancer cell lines, DU145 and 22RV1, generates a subtle reduction in colony formation that was further reduced in combination with knockdown of *NOTCH2* [135].

Hh signaling was found to regulate the anti-apoptotic molecule, Bcl-2, in docetaxel-resistant PCSCs, and treatment with the Bcl-2 inhibitor, ABT-737, reduced colony formation in PCSCs, recapitulating the effect observed with Hh pathway inhibition [135]. Additionally, intraprostatic injection of pCX-Shh-IG-GFP vectors in mice, resulting in persistent Shh ligand overexpression in adult prostates, leads to the development of invasive and metastatic prostate cancers within 90 days [136]. In this model, it was also found that active Hh signaling was localized to p63-expressing prostate stem cells, demonstrating that in addition to breast cancer, p63 also drives Hh signaling in PCSCs. Furthermore, the progeny of p63-expressing PCSCs conveyed the ability to differentiate into cells of a basal-intermediate and intermediate-luminal phenotype, as well as rare ChgA^+^ neuroendocrine cells [136]. Lastly, Hh pathway blockade by GANT61 or genistein, an isoflavone constituent in soybeans, was able to suppress tumoursphere formation and colony formation, further implicating the pathway in prostate CSC maintenance [133].

### 4.11. Lung Cancer

Small cell lung cancer (SCLC), representing approximately 20%–25% of all lung tumours, is a highly aggressive and lethal malignancy with a five-year survival rate of 2%–8%. SCLC is an excellent example of how stem/progenitor cells escape from niche-dependent signals via constitutive Hh pathway activation [176,177]. Human SCLC cell lines are characterized by the expression of many genes associated with early developmental and progenitor cell states, such as BMP4, normally required during lung epithelial development, Nestin and ASH-1, a transcription factor required for pulmonary neuroendocrine differentiation [82]. Importantly, Hh pathway inhibition by cyclopamine inhibits the expression of all three genes [82].

Deletion of *Smo* in a genetic mouse model of SCLC significantly reduced tumour initiation and progression, whereas mice expressing SmoM2 developed more frequent and considerably larger tumours [121]. Tumour cells isolated from the same genetic mouse model of SCLC crossed with a Ptch1^LacZ/+^ reporter mouse express *LacZ*, indicating that SCLCs maintain active Hh signaling autonomously. Inhibition of Hh signaling in the human SCLC cell line, LX22CL, using LDE-225, shSMO or 5E1, resulted in fewer colonies in a colony formation assay. Conversely, pathway activation using adenovirally-expressed SmoM2 or recombinant Shh protein increased clonogenicity [121]. Interestingly, LX22CL cells surviving a single round of carboplatin and etoposide were considerably more sensitive to pathway manipulation in the same assay. While the growth of chemonaive LX22 xenografts in mice was largely unaffected by treatment with the Hh inhibitor LDE-225, a combination therapy of a round of carboplatin and etoposide followed by LDE-225 treatment prevented tumour relapse, suggesting that the ability of chemoresistant SCLC cells to regenerate is dependent on Hh signaling. Furthermore, LX22 xenografts displayed a marked increase in Shh ligand expression and Gli2 nuclear localization post chemotherapy *in vivo*, and the proportion of cells expressing a primary cilium increased from less than 1% to approximately 20% [121]. Together, these data suggest a critical role for the Hh pathway in CSC maintenance in SCLC and reveal that Hh inhibitors and chemotherapy may be an effective combination therapy. Similarly, the CSC side population of the SCLC cell line, H1339, detected by the ability to expel Hoechst stain from active ABCG2 transporters, was significantly reduced from 0.75%, following cisplatin treatment alone, to 0.18% when treated with both cisplatin and the Smo inhibitor GDC-0449 [122]. Taken together, active Hh signaling promotes a chemoresistant phenotype in SCLC, and Hh pathway inhibition sensitizes CSCs to cytotoxic therapy and prevents tumour relapse [121].

Lung squamous cell carcinoma (LSCC) exhibits cell-autonomous Hh pathway activation through a protein kinase Ci (PKCi)-SOX2-Hh signaling axis to maintain a CSC-like phenotype in lung oncospheres [103,178]. PKCi mediates SOX2 recruitment to the *HHAT* promoter to induce constitutive Hh ligand production. These oncospheres are characterized by *SOX2*, *OCT4*, *NANOG* and *ALDHA1* mRNA expression, high colony formation efficiency, enhanced growth in soft agar and enhanced tumourigenic potential *in vivo* that recapitulates the parental tumour [103]. Additionally, inhibition of Hh signaling via *siSHH* or GDC-0449 treatment in CSCs derived from the lung adenocarcinoma (LAC) cell line A549M sensitized to erlotinib and cisplatin treatment [123]. Similarly, treatment of ABCG2 expressing CSCs in LAC cell line HCC, with both cisplatin and GDC-0449, dramatically decreased the fraction of surviving cells [122], indicating that combination therapy can effectively inhibit tumour growth, compared to either treatment alone.

### 4.12. Melanoma

Characterized as the most aggressive and lethal skin cancer with high metastatic potential, enhanced heterogeneity and resistance to chemotherapy, advanced metastatic melanoma has a poor prognosis with a median survival time of 6–9 months and a three-year survival rate of 10%–15% [137,179]. A large body of evidence suggests that within the heterogeneous population that constitutes the tumour bulk, active Hh signaling maintains a subpopulation of CSCs [137,179,180]. Cultured CSCs melanospheres demonstrate the Hh-driven CSC properties of increased levels of pluripotency factors, *SOX2*, *NANOG*, *OCT4* and *KLF4*, and Hh pathway components, *SHH*, *PTCH1*, *SMO*, *GLI2*, *GLI3*, high ALDH activity, the ability to clonally expand *in vitro* and initiate tumours representing the primary tumour *in vivo* [137].

Knockdown of both *SMO* and *GLI1* in SSM2c and A375 melanospheres and engraftment of SSM2c cells transduced with lentiviral-shSMO and LV-ShGLI1 leads to a drastic decrease in the fraction of ALDH^+^ cells, reduced clonogenicity and reduced tumour growth, respectively [137]. Furthermore, knockdown of SMO leads to a complete abolishment of *SOX2* mRNA, suggesting that SOX2 is a downstream mediator of the Hh signaling pathway in melanoma CSCs. Indeed, ChIP sequencing in M26c melanoma CSCs demonstrated that *SOX2* is a direct transcriptional target of GLI1 and GLI2 [138]. Additionally, WIP1, an oncogenic phosphatase overexpressed in several types of human cancer [181,182,183,184,185], is required for Hh-induced melanoma CSC growth and self-renewal. WIP1 knockdown in SSM2c melanospheres decreased endogenous *Gli1* expression and diminished the increased colony formation potential induced by *shPTCH1* [139].

## 5. Targeting Hedgehog Signaling in Cancer Stem Cells

Like normal somatic stem cells, CSCs are resistant to conventional chemotherapeutics, primarily due to the expression of drug efflux pumps and a reduced replication rate. As a result, residual CSCs represent a significant hurdle in the prevention of disease recurrence and metastatic spread. Evidence described above in CML, AML, GBM, gastric cancer, pancreatic cancer, prostate cancer, SCLC and LSCC clearly demonstrates that targeting the Hh signaling pathway in CSCs sensitizes these cells to cytotoxic drug and radiation-mediated cell death and reduces self-renewal potential, leading to abolishment or reduced tumour relapse (Table 1).

Compelling evidence suggests that inhibition of Hh signaling in CSCs promotes commitment or differentiation and a loss of “stemness”, as supported by a reduction in clonogenicity and pluripotency markers, thereby limiting the characteristics normally supporting chemoresistance. Therefore, the combinatorial targeting of CSCs and tumour bulk with Hh inhibitors and conventional chemotherapeutics and/or radiation is an attractive approach to prevent tumour relapse and maximize patient outcomes. However, further investigation into the sequencing of Hh inhibitors and conventional therapies is required to determine whether priming CSCs prior to cytotoxic treatment, co-administration and/or as maintenance therapy following tumour debulking will lead to optimal outcomes. Importantly, the type of Hh antagonist for individual cancer subtypes must also be carefully considered based on the mode of pathway activation.

## 6. Conclusions

The inability to fully eradicate CSCs is a significant clinical problem and leads to tumour recurrence, therapy resistance and metastatic spread of disease. In this review, we have highlighted the critical role of a key embryonic signaling pathway, the Hh pathway, in the maintenance of CSCs in a number of haematological malignancies and solid tumours. Mounting evidence suggests that targeting the Hh signaling pathway in CSCs may provide a viable and efficacious clinical option to limit tumour growth, overcome resistance and prevent disease relapse. However, a greater understanding of Hh-mediated CSC maintenance and how to best combine Hh antagonists with conventional therapies in the clinic will be required before the full potential of this possibility is realized.
